# Threshold effect of exercise frequency on comprehensive physical fitness with sex-specific mediation by body mass index in Chinese university students

**DOI:** 10.3389/fpubh.2026.1954509

**Published:** 2026-10-28

**Authors:** Yuehui Zhao, Ya Zhao, Yuanzhen Wang

**Affiliations:** 1 School of Physical Education, Yangzhou University, Yangzhou, China; 2 The International Development and Cooperation Institute, Hiroshima University, Higashihiroshima, Japan

**Keywords:** body mass index, exercise frequency, physical fitness, sex-specific mediation, university students

## Abstract

**Background:**

University students represent a critical population for national health, yet the dose-response relationship between objectively measured exercise frequency and comprehensive physical fitness, along with the mediating role of body composition, remains insufficiently characterized in this demographic.

**Objective:**

This study aimed to systematically investigate the associations between exercise frequency and multi-dimensional physical fitness indicators and to examine the sex-specific mediating role of body mass index (BMI) in a large cohort of Chinese undergraduates.

**Methods and results:**

A cross-sectional analysis was conducted among 15,100 university students (7,653 males) using exercise frequency data automatically recorded by the Chuanggao-Sports mobile application. Participants were categorized into low (≤ 2 sessions/week), moderate (3–4 sessions/week), and high (≥5 sessions/week) frequency groups. Analysis of variance revealed a pronounced threshold-like associational pattern, wherein transitioning from low to moderate frequency yielded significant improvements in the overall fitness score and most specific indicators, while no statistically detectable additional fitness gains were observed in this sample when moving from moderate-to-high exercise frequency. To further objectively identify this threshold, we performed a restricted cubic spline (RCS) analysis. The RCS model demonstrated a significant non-linear relationship between weekly exercise frequency and predicted physical fitness score (*P* for non-linearity < 0.0001). The bootstrap resampling (1,000 iterations) identified a threshold (inflection point) at 2.01 sessions/week (95% CI: 2.00 to 4.00), with a statistically significant change in slope. Sex-stratified path analysis further demonstrated distinct associational pathways: In males, exercise frequency showed both a significant positive association with physical fitness and an indirect associational pattern linked to BMI. In females, exercise frequency maintained a significant direct positive association with physical fitness, but the indirect association pattern through BMI was not supported. All reported relationships represent statistical associational patterns; reverse causality is plausible given the cross-sectional study design. Note that sex-specific associational-mediation results derived from path-analysis should be treated as purely exploratory given sub-optimal model-data fit.

**Discussion:**

This study provides empirical evidence for a threshold-like associational pattern of exercise frequency on physical fitness and exploratory observations of sex-specific associational-mediation patterns based on BMI, a crude proxy of body composition.The findings underscore that pragmatic campus interventions should prioritize shifting inactive students to a moderate activity level, with sex-tailored strategies focusing on BMI management for males while exploring non-BMI pathways for females.

## Introduction

University students are at a critical developmental stage where physical functions continue to mature and lifestyle habits consolidate, making their physical fitness a direct determinant of the future health quality of the national populace. Research consistently indicates that the physical health of this population is influenced by a complex interplay of behavioral and environmental factors, with unhealthy patterns such as breakfast skipping linked to reduced muscle strength and sprint performance (1) and the pervasive effects of the COVID-19 pandemic significantly impairing cardiorespiratory endurance and contributing to weight gain (2, 3). A substantial body of cross-sectional and longitudinal evidence has established that regular physical exercise is a critical determinant of multiple fitness dimensions, including cardiorespiratory endurance, muscular strength, and flexibility (4). For instance, students who actively participate in exercise have a significantly lower risk of obesity and perform better in grip strength and lower limb power tests compared to sedentary peers (4) and structured exercise prescription interventions improve performance in cardiorespiratory fitness tests like the 12-min run and anaerobic power metrics such as the standing long jump and 50-meter dash (5). Despite these well-recognized benefits of exercise on overall physical performance, the specific dose-response relationship between varying exercise frequencies and a comprehensive battery of fitness indicators, as well as the mediating pathways through body composition, remains insufficiently explored in this demographic. While previous work has examined associations between physical fitness and mental health (6) or the impact of parenting styles (7) a mechanistic understanding of how varying exercise frequencies influence a broad spectrum of fitness indicators is lacking. Prior research has frequently employed a dichotomous approach comparing “exercisers” vs. “non-exercisers,” which may obscure nuanced dose-response relationships, and has predominantly focused on fixed training frequencies—comparing, for example, two vs. three sessions per week—yielding heterogeneous results. Some studies report that increasing frequency from two to three sessions per week confers additional benefits for specific components like change-of-direction speed (8) while others suggest overall fitness improvements are largely independent of frequency provided a minimum threshold is met (8, 9). For instance, multicomponent training performed two or three times weekly produced similar improvements in body composition and aerobic capacity in obese children (9) and concurrent exercise training three vs. five times per week led to comparable increases in fitness in older adults (10). This raises the unresolved question of whether a threshold effect exists where fitness benefits plateau after a certain frequency is reached, with some evidence indicating that even once-weekly physical activity yields substantial benefits compared to inactivity (11) and a meta-analysis highlighting three sessions per week for at least 30 min as effective without systematically examining higher frequencies. Crucially, the possibility that increasing exercise frequency from three to five sessions per week may not confer additional significant improvements—a phenomenon with important public health implications—has not been systematically tested in general university students.

The physiological pathways linking physical exercise to physical fitness in young adults, particularly whether changes in fitness indicators involve mediational associations related to favorable changes in body composition, remain insufficiently supported by empirical evidence. While the benefits of regular activity for metabolic health and functional capacity are broadly recognized, specific causal pathways—such as the role of body fat reduction or lean mass accretion—are often inferred from studies in older or clinical populations (12). In healthy young cohorts, where body composition is generally more labile and adaptive to training stimuli, the mediating effect of body composition on fitness outcomes has not been rigorously established using objective, large-scale data. The relationship between physical activity, body composition, and physical fitness is complex, with body mass index (BMI) often posited as a potential intermediary. Some evidence suggests higher BMI is associated with diminished exercise capacity and blunted physiological responses, such as reduced heart rate variability recovery (13, 14) and obesity negatively influences acute cognitive benefits from exercise (15) while elevated BMI in older adults may counteract some beneficial effects on strength (16). However, other findings indicate that benefits of exercise on health outcomes can be observed regardless of baseline BMI, suggesting the role of BMI as a mediator is not universally consistent (17, 18) and the impact of exercise on body weight itself is variable, influenced by baseline BMI and exercise type (19). Furthermore, sex-specific differences in body composition responses to exercise, driven by distinct hormonal and physiological profiles, are frequently overlooked in existing research, limiting generalizability across genders (20, 21). Research on exercise motivation and physical activity levels affects fitness differently in male and female students (22) and the association between BMI and physical fitness index is non-linear and varies by sex (23) with a cross-sectional study from Yangzhou demonstrating that fitness decline associated with higher BMI is more pronounced in males (24). Most existing investigations have employed pooled analyses or single-group designs, limiting the ability to delineate sex-specific mechanistic pathways, and comparative path analyses separately constructed for male and female students are scarce, leaving a critical gap regarding sex-differentiated mediation effects.

Methodologically, prior investigations have frequently relied on self-reported physical activity data, which is susceptible to recall bias and social desirability effects, compromising validity. This reliance leads to misclassification and attenuated effect estimates, underscoring the need for objective measurement tools such as mobile applications to collect precise exercise behavior data. Recent advancements in mobile health (mHealth) technology provide novel opportunities for objective monitoring, capturing granular, real-time data with minimal participant burden. To address these gaps, the present study employed a large-scale cross-sectional design, utilizing objectively recorded exercise frequency from a mobile sports application to systematically compare differences across multiple physical fitness indicators among Chinese university students. This approach allows for more precise examination of associations between habitual exercise frequency and various fitness dimensions, and innovatively constructs a sex-stratified path analysis model to examine both the direct associational pattern of exercise frequency with physical fitness and the indirect associational pattern potentially linked to BMI. Accordingly, this study aimed to investigate the differences in physical fitness across distinct exercise frequency groups and to explore the potential associational mediating pattern of BMI, with the goal of providing evidence-based guidance for targeted physical activity interventions in higher education settings.

## Materials and methods

### Ethics statement

This study was conducted in accordance with the Declaration of Helsinki and approved by the Institutional Review Board of Yangzhou University (Approval No. YXYLL-2025-152). Written informed consent was obtained from all participants prior to data collection.

### Study design and participants

This study employed a cross-sectional observational design. Participants were recruited using a random sampling method from the undergraduate student population of a Chinese university. Exercise frequency was monitored through the “Chuanggao Sports” app, where engaging in physical activity for more than 30 min was considered one valid exercise session. A total of 16,538 students were initially recruited and invited to participate. Accordingly, 1,438 participants were excluded due to missing key-variable data. Following data quality screening, participants with missing values on any of the key variables were excluded from the analysis. Complete-case analysis was adopted in the present study. Formal testing for missing-completely-at-random is not feasible in this dataset, so selection bias driven by non-random missingness cannot be excluded. Baseline characteristics for excluded participants were not archived and cannot be compared against included samples. After this exclusion procedure, the final analytical sample comprised 15,100 undergraduate students, consisting of 7,653 males and 7,447 females.

Based on the exercise frequency automatically collected by the Chuanggao Sports mobile app, participants were classified into three ordinal levels. The low-frequency group, defined as engaging in two or fewer exercise sessions per week, comprised 2,446 students, representing 16.20 percent of the final analytical sample. This group included 1,711 males and 735 females. The moderate-frequency group, defined as engaging in three to four exercise sessions per week, comprised 9,475 students, representing 62.75 percent of the final analytical sample, with 4,310 males and 5,165 females. The high-frequency group, defined as engaging in five or more exercise sessions per week, comprised 3,179 students, representing 21.05 percent of the final analytical sample, including 1,632 males and 1,547 females (Table 1).

**Table 1:** Basic attributes of the sample.

Exercise frequency	Male	Female	Overall
Low	1,711	735	2,446
Moderate	4,310	5,165	9,475
High	1,632	1,547	3,179

All participants in the final analytical sample underwent a standardized physical fitness assessment battery. The male subsample completed seven fitness indicators plus pull-up assessment, while the female subsample completed seven fitness indicators plus sit-up assessment. It should be noted that pull-up and sit-up tests are not equivalent measurements. Multi-group measurement invariance testing was not performed in the current study.

### Measures

#### Exercise frequency

Exercise frequency data were collected objectively via a mobile fitness application named Chuanggao Sports, a widely used campus sports APP among Chinese university students. The APP automatically recorded participants' exercise sessions, including date, time, and duration of each workout. It should be noted that only the frequency of valid sessions was recorded in the present dataset, while objective information regarding exercise intensity was not available. Restricted cubic spline regression showed a non-linear association between weekly exercise frequency and physical fitness score. Physical fitness slightly decreased when weekly exercise frequency was below 2 times, and then increased rapidly, reaching a peak at approximately 4 times per week, followed by a gradual decline (Figure 1). The threshold for the maximum change in effect magnitude was 2.00 times per week (95%CI: 2.00–4.00). The relatively wide bootstrap confidence interval highlights substantial uncertainty around this estimated inflection point. Based on this, and the number of exercise sessions recorded by the sports APP, participants were classified into three ordinal levels: low frequency (≤ 2 sessions per week), moderate frequency (3–4 sessions per week), and high frequency (≥5 sessions per week). It should be highlighted that exercise frequency alone cannot fully reflect total exercise dose. Participants with identical weekly exercise frequency may have substantially different actual training loads, since objective data regarding exercise intensity, modality and exact single-session duration are not available in this dataset.

**Figure 1:**
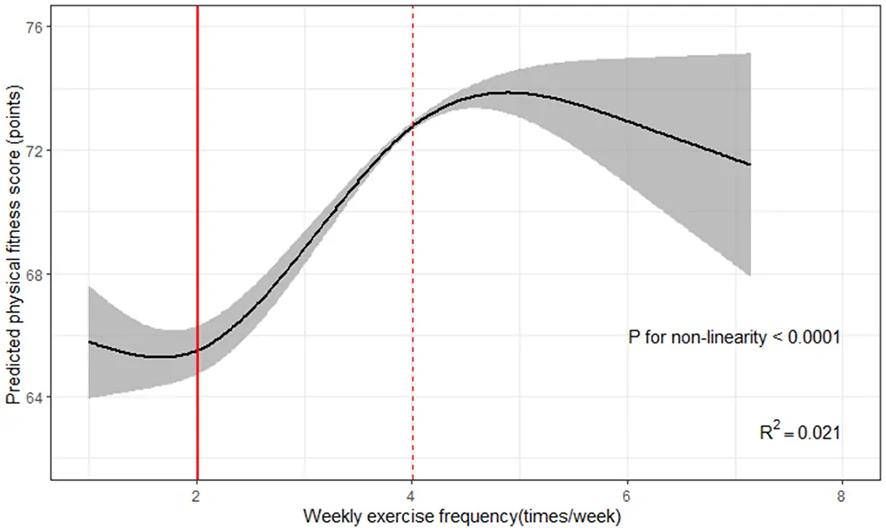
Non-linear relationship between weekly exercise frequency and predicted physical fitness score. The solid line shows the predicted score, and the shaded area indicates the 95% confidence interval (CI). The red line indicates the threshold at 2.00 times/week (*P* for non-linearity < 0.0001, *R*^2^ = 0.021).

The restricted cubic spline model yielded R^2^ = 0.021, indicating that weekly exercise frequency alone accounted for approximately 2.1% of the variance in the predicted overall physical fitness score. Although the non-linear association was highly statistically significant (*P* for non-linearity < 0.0001), this significance should be interpreted in light of the very large sample size (*N* = 15,100). The modest R^2^ value is biologically expected: physical fitness is a multi-determined trait jointly shaped by genetic endowment, body composition, nutritional status, sleep quality, habitual training history, and other lifestyle factors, of which exercise frequency is only one single behavioral exposure. Accordingly, exercise frequency by itself constitutes a relatively weak predictor of overall physical fitness. The statistically detectable non-linear association should therefore be regarded as a real but modest association, rather than evidence that exercise frequency is a strong determinant of fitness. This observation further reinforces our cautious, associational interpretation of the present cross-sectional findings.

#### Body composition

Body Mass Index (BMI) was calculated from measured height and weight and expressed as a standardized BMI score. BMI served as the mediator variable (M) in the path analysis model. It should be noted that BMI is a crude proxy for body composition and cannot differentiate fat mass from lean mass. More detailed body-composition indicators such as body fat percentage and waist circumference were not available in the present dataset.

#### Physical fitness indicators

Physical fitness was evaluated using a comprehensive battery of standardized tests. For body composition, BMI score was assessed. Cardiorespiratory fitness was measured by vital capacity score. Lower limb muscular strength was evaluated using standing long jump score. Flexibility was assessed via sit-and-reach score. Speed was measured by 50-meter dash score. Endurance was evaluated using endurance run score. Upper limb and core strength were assessed with sex-specific tests: males completed the pull-up test, whereas females completed the sit-up test. In addition, an overall fitness score was computed to reflect comprehensive physical fitness status. All fitness scores were standardized to facilitate comparison across domains and groups.

The latent variable “physical quality” was constructed from a set of routine national-student-fitness-test observed indicators including dash score, vital-capacity score, standing-long-jump score, sit-and-reach score, endurance-run score, and sex-specific strength-test scores (pull-ups for males, sit-ups for females).

### Statistical analysis

#### Descriptive and inferential statistics

All main analyses presented in this manuscript report unadjusted crude associations; key potential confounding covariates were not collected and could not be included in analytical models. Descriptive statistics were reported as mean ± standard deviation (SD) for continuous variables. One-way analysis of variance (ANOVA) was used to examine overall differences in physical fitness scores across the three exercise frequency groups. When the ANOVA yielded a statistically significant result (*p* < 0.05), Tukey's Honestly Significant Difference (HSD) *post-hoc* test was applied to identify pairwise differences between groups. Statistical significance was set at *p* < 0.05. Restricted cubic spline (RCS) regression was performed using the rms package in R to model the non-linear association between weekly exercise frequency and overall physical fitness score. We intended to place four knots at the 5th, 35th, 65th, and 95th percentiles of weekly exercise frequency. However, duplicate knot values occurred due to the discrete-integer nature of weekly exercise frequency (range: 0-7 sessions per week), which caused model convergence failure. Therefore, four pre-specified knots at weekly exercise frequency of 1, 2, 4, and 6 sessions were adopted for the final analysis, covering our a-priori grouping cut-offs (≤ 2, 3–4, ≥5 sessions/week). To identify the threshold with the maximum magnitude of slope change, we computed first-order derivatives of spline-predicted fitness values across a dense sequence of weekly exercise-frequency values (*n* = 300 equally spaced points). The position with the largest absolute change in slope was defined as the threshold. The 95 % confidence interval for this threshold was derived via 1,000-time bootstrap resampling. Analyses were run under R4.3.1, with rms 6.7-1 and ggplot 2-3.4.4 packages for spline and visualization work. Individual fitness test scores were standardized using z-score transformation. The composite overall-fitness score was computed based on standardized sub-dimension fitness indicators.

Residual diagnostics were performed for all one-way ANOVA models. The male pull-up score and, to a lesser extent, the female sit-up score showed marked positive skewness, with residual distributions moderately deviating from normality. Given the very large group sample sizes (each group comprising over 1,600 students), the Central Limit Theorem supports the robustness of one-way ANOVA and Tukey HSD *post-hoc* comparisons of group means against moderate departures from normality. Effect-size estimates (partial η^2^) are reported in Table 2 to complement *p*-values, so that readers can evaluate practical relevance alongside statistical significance.

**Table 2:** Component difference analysis of each measurement index.

Exercise Frequency	Mean ±SD	*F*-value	*p*-value	pes	Tukey HSD	*N*
Body composition (BMI Score)						
Low	89.01 ± 14.35	57.11	*P* < 0.001	0.008	0.4770483	15,100
Moderate	92.14 ± 12.69				*P* < 0.001	
High	91.83 ± 12.91				*P* < 0.001	
Cardiorespiratory fitness (Vital Capacity Score)						
Low	78.17 ± 15.24	3.11	0.0445	0.000412	0.9996363	15,100
Moderate	79.02 ± 15.39				0.1040196	
High	79.01 ± 15.46				0.0394234	
Lower limb strength (Standing Long Jump Score)						
Low	61.84 ± 21.53	16.21	*P* < 0.001	0.002	0.9269020	15,100
Moderate	64.3 ± 19.57				*P* < 0.001	
High	64.45 ± 19.37				*P* < 0.001	
Upper limb Strength (Pull-up Score)						
Low	14.97 ± 25.44	9.43	*P* < 0.001	0.002	0.5017742	7,653
Moderate	17.89 ± 27.98				*P* < 0.001	
High	18.79 ± 28.51				*P* < 0.001	
Core strength (Sit-up Score)						
Low	68.87 ± 15.56	3.9	0.0203	0.001	0.8896355	7,447
Moderate	70.36 ± 14.19				0.0240971	
High	70.55 ± 14.46				0.0228855	
Flexibility (Sit-and-Reach Score)						
Low	73.76 ± 16.46	87.9	*P* < 0.001	0.012	0.6732257	15,100
Moderate	78.06 ± 14.74				*P* < 0.001	
High	78.32 ± 14.16				*P* < 0.001	
Speed (50m Dash Score)						
Low	75.88 ± 13.39	5.94	0.00264	0.000786	0.1502885	15,100
Moderate	74.99 ± 11.69				0.3833309	
High	75.45 ± 11.97				0.0033344	
Endurance (Endurance Run Score)						
Low	61.14 ± 18.16	187.57	*P* < 0.001	0.024	0.8990151	15,100
Moderate	67.86 ± 15.26				*P* < 0.001	
High	68.01 ± 15.49				*P* < 0.001	
Overall fitness score						
Low	69.15 ± 9.31	210.7	*P* < 0.001	0.027	0.7195929	15,100
Moderate	73.12 ± 8.54				*P* < 0.001	
High	72.99 ± 8.65				*P* < 0.001	

#### Path analysis

To investigate the statistical associations among exercise frequency, BMI, and physical fitness, a path analysis model was constructed using structural equation modeling (SEM). The model was tested separately for male (*n* = 7,653) and female (*n* = 7,447) samples to account for sex-specific differences in fitness indicators.

The model specification was as follows. Exercise frequency was designated as the exogenous variable (X). BMI served as the mediator variable (M). Physical quality was modeled as an endogenous latent variable estimated from observed fitness indicators. The observed indicators included dash score, vital capacity score, standing long jump score, sit-and-reach score, endurance run score, and pull-up score for the male sample, and dash score, vital capacity score, standing long jump score, sit-and-reach score, endurance run score, and sit-up score for the female sample. The factor loading of physical quality on dash score was fixed to 1.0 to set the scale of the latent variable. Model parameters were estimated using unstandardized path coefficients with standard errors (SE), critical ratios (CR), and *p*-values. Standardized estimates were also reported to facilitate interpretation of effect sizes.

Model fit was evaluated using multiple indices, with the following acceptable and excellent thresholds applied: Goodness-of-Fit Index (GFI) greater than 0.90 for acceptable fit and greater than 0.95 for excellent fit; Adjusted Goodness-of-Fit Index (AGFI) greater than 0.90 for acceptable fit and greater than 0.95 for excellent fit; Comparative Fit Index (CFI) greater than 0.90 for acceptable fit and greater than 0.95 for excellent fit; and Root Mean Square Error of Approximation (RMSEA) less than 0.08 for acceptable fit and less than 0.05 for excellent fit. The chi-square to degrees-of-freedom ratio (χ^2^/df) was also reported.

All statistical analyses were performed using appropriate statistical software packages. Basic data sorting was performed in Microsoft Excel. R software (rms, ggplot2 packages) was used for descriptive statistics, one-way analysis of variance, Tukey's HSD *post-hoc* test and restricted cubic spline analysis. Path analysis via structural equation modeling was carried out using AMOS. Significance levels were denoted as *p* < 0.05, *p* < 0.01, *p* < 0.001, and non-significant (ns).

## Results

### Differences in physical fitness across exercise frequency groups

The descriptive statistics and one-way ANOVA results for all physical fitness indicators across the three exercise frequency groups are presented below. The low-frequency group consisted of students engaging in two or fewer exercise sessions per week, the moderate-frequency group engaged in three to four sessions per week, and the high-frequency group engaged in five or more sessions per week.

For body composition, as measured by BMI score, the low-frequency group demonstrated a mean score of 89.01 with a standard deviation of 14.35. The moderate-frequency group showed a higher mean score of 92.14 with a standard deviation of 12.69, and the high-frequency group exhibited a mean score of 91.83 with a standard deviation of 12.91. The one-way ANOVA revealed a highly significant overall difference across the three groups, with an F-value of 57.11 and a *p*-value less than 0.001 (Table 2).

Regarding cardiorespiratory fitness, as assessed by vital capacity score, the low-frequency group achieved a mean score of 78.17 with a standard deviation of 15.24. The moderate-frequency group attained a mean of 79.02 with a standard deviation of 15.39, while the high-frequency group recorded a mean of 79.01 with a standard deviation of 15.46. The overall group difference reached marginal statistical significance, yielding an F-value of 3.11 and a *p*-value of 0.045 (Table 2).

For lower limb strength, evaluated via standing long jump score, the low-frequency group presented a mean score of 61.84 with a standard deviation of 21.53. The moderate-frequency group demonstrated a mean of 64.30 with a standard deviation of 19.57, and the high-frequency group showed a mean of 64.45 with a standard deviation of 19.37. The ANOVA indicated a statistically significant overall difference, with an F-value of 16.21 and a *p*-value less than 0.001 (Table 2).

In the assessment of upper limb strength using pull-up score among the male subsample of 7,653 participants, the low-frequency group displayed a mean score of 14.97 with a standard deviation of 25.44. The moderate-frequency group achieved a mean of 17.89 with a standard deviation of 27.98, and the high-frequency group recorded a mean of 18.79 with a standard deviation of 28.51. The overall difference across groups was statistically significant, with an F-value of 9.43 and a *p*-value less than 0.001 (Table 2).

For core strength, measured by sit-up score among the female subsample of 7,447 participants, the low-frequency group exhibited a mean score of 68.87 with a standard deviation of 15.56. The moderate-frequency group showed a mean of 70.36 with a standard deviation of 14.19, and the high-frequency group demonstrated a mean of 70.55 with a standard deviation of 14.46. The ANOVA yielded an F-value of 3.90 with a *p*-value of 0.020, indicating a statistically significant overall difference (Table 2).

Flexibility, as measured by sit-and-reach score, revealed substantial group differences. The low-frequency group obtained a mean score of 73.76 with a standard deviation of 16.46. The moderate-frequency group achieved a considerably higher mean of 78.06 with a standard deviation of 14.74, and the high-frequency group recorded a mean of 78.32 with a standard deviation of 14.16. This domain exhibited the largest F-value among all indicators, at 87.90, with a *p*-value less than 0.001 (Table 2).

For speed, assessed by 50-meter dash score, the low-frequency group presented a mean of 75.88 with a standard deviation of 13.39. The moderate-frequency group showed a mean of 74.99 with a standard deviation of 11.69, and the high-frequency group demonstrated a mean of 75.45 with a standard deviation of 11.97. The ANOVA produced an F-value of 5.94 with a *p*-value of 0.003, indicating statistical significance (Table 2).

Endurance, evaluated through endurance run score, displayed pronounced group differences. The low-frequency group exhibited a mean score of 61.14 with a standard deviation of 18.16. The moderate-frequency group achieved a substantially higher mean of 67.86 with a standard deviation of 15.26, and the high-frequency group recorded a mean of 68.01 with a standard deviation of 15.49. This domain yielded the second-highest F-value at 187.57, with a *p*-value less than 0.001 (Table 2).

For overall fitness score, the low-frequency group demonstrated a mean of 69.15 with a standard deviation of 9.31. The moderate-frequency group showed a mean of 73.12 with a standard deviation of 8.54, and the high-frequency group exhibited a mean of 72.99 with a standard deviation of 8.65. The ANOVA revealed the highest F-value across all comparisons at 210.70, with a *p*-value less than 0.001 (Table 2).

Tukey HSD *post-hoc* analyses revealed a consistent pattern across the majority of fitness indicators. For body composition, lower limb strength, upper limb strength among males, core strength among females, flexibility, endurance, and overall fitness, the low-frequency group scored significantly lower than both the moderate-frequency and high-frequency groups. However, no significant differences were detected between the moderate-frequency and high-frequency groups for any of these indicators. For cardiorespiratory fitness and speed, only the comparison between the low-frequency and moderate-frequency groups reached statistical significance, whereas the comparison between the low-frequency and high-frequency groups did not. Notably, across all nine fitness domains examined, no statistically significant differences emerged between the moderate-frequency and high-frequency exercise groups, suggesting a threshold-like associational pattern wherein increasing exercise frequency from low to moderate yields significant fitness benefits, whereas further increases from moderate to high do not produce additional statistically significant improvements in this sample. Note that these are unadjusted group-level differences without adjustment for sociodemographic and lifestyle confounding factors. Given the large sample size, statistical significance does not necessarily correspond to practically meaningful differences; partial η^2^ effect-size estimates are provided in Table 2 to aid interpretation of real-world relevance.

### Visual representation of group differences

The boxplot visualization of physical fitness scores across exercise frequency groups depicted nine separate panels arranged in a three-by-three grid format, corresponding to the nine fitness indicators assessed. Each panel displayed three boxplots representing the low, moderate, and high exercise frequency groups, color-coded in red, green, and blue respectively (Figure 2).

**Figure 2:**
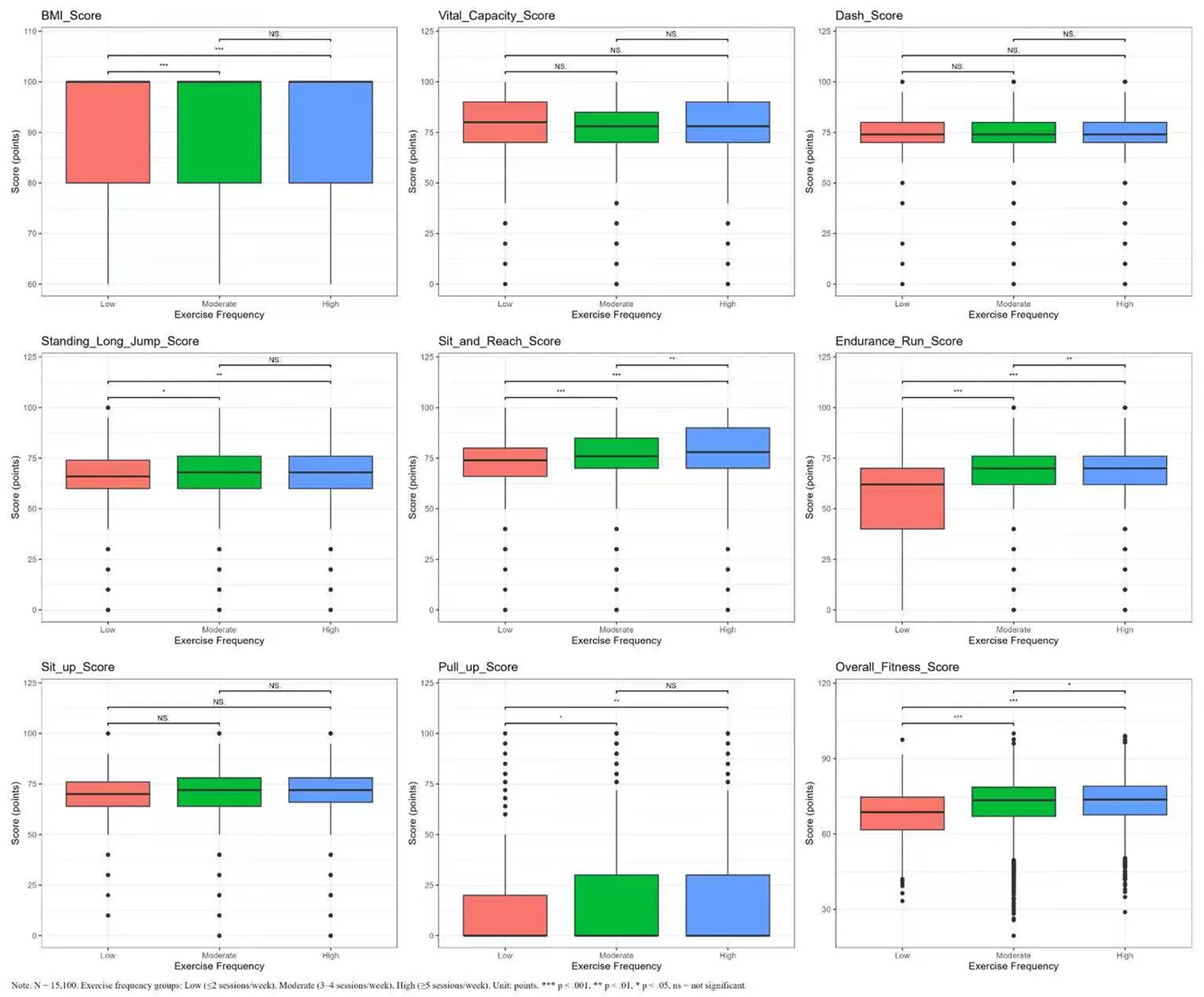
Boxplots of physical fitness scores across exercise frequency groups. The 3 × 3 grid represents the nine assessed fitness indicators. Within each panel, the red, green, and blue boxplots correspond to the low, moderate, and high exercise frequency groups, respectively.

In the BMI score panel, the low-frequency group exhibited a median value near 90 with a relatively wide interquartile range extending from approximately 80 to 100. The moderate-frequency group showed a slightly higher median near 93 with a compressed interquartile range, while the high-frequency group displayed a median comparable to the moderate group. Statistical annotation indicated significant differences between the low and moderate groups as well as between the low and high groups, denoted by triple asterisks, whereas no significant difference was observed between the moderate and high groups, marked as non-significant.

The vital capacity score panel revealed overlapping distributions across all three groups, with medians clustering near 80. The low-frequency group showed a median slightly below 80, while both the moderate and high groups exhibited medians near 80 to 82. Statistical annotations indicated a significant difference only between the low and moderate groups, denoted by a single asterisk, with all other pairwise comparisons marked as non-significant.

For the dash score panel, the low-frequency group displayed a median near 76 with the moderate group showing a slightly lower median near 75 and the high group near 75 as well. Significant differences were noted between the low and moderate groups, denoted by double asterisks, while comparisons involving the high group were non-significant.

The standing long jump score panel showed the low-frequency group with a median near 62 and a wide interquartile range spanning approximately 50 to 75. Both the moderate and high groups exhibited higher medians near 65 with somewhat narrower distributions. Significant differences were observed between the low group and both the moderate and high groups, denoted by triple asterisks, with no significant difference between the moderate and high groups.

The sit-and-reach score panel demonstrated the most pronounced visual separation among groups. The low-frequency group showed a median near 74 with an interquartile range from approximately 65 to 85. The moderate group exhibited a markedly higher median near 79, and the high group showed a comparable median near 79 with a slightly tighter distribution. All pairwise comparisons except the moderate vs. high comparison reached significance at the triple asterisk level.

The endurance run score panel revealed substantial differences, with the low-frequency group showing a median near 62 and a broad interquartile range extending from approximately 50 to 75. The moderate and high groups both displayed higher medians near 68 with narrower distributions. Significant differences were observed between the low group and both other groups, denoted by triple and double asterisks, respectively, with no significant difference between the moderate and high groups.

The sit-up score panel, representing female participants, showed modest differences with the low-frequency group median near 69 and the moderate and high groups both near 71. The low vs. moderate comparison reached significance at the single asterisk level, as did the low vs. high comparison, while the moderate vs. high comparison was non-significant.

The pull-up score panel, representing male participants, displayed highly skewed distributions with the low-frequency group showing a median near 10 and a very wide range extending from near zero to approximately 80. The moderate group exhibited a higher median near 20, and the high group showed a comparable median with a similarly wide distribution. Significant differences were observed between the low group and both the moderate and high groups at the triple asterisk level, with no significant difference between the moderate and high groups.

The overall fitness score panel showed the low-frequency group with a median near 70 and an interquartile range from approximately 65 to 75. Both the moderate and high groups exhibited higher medians near 73 with tighter distributions. Significant differences were detected between the low group and both other groups at the triple asterisk level, with the moderate vs. high comparison marked as non-significant.

### Path analysis: model fit

Separate path analysis models were estimated for male and female samples to examine the statistical associations of exercise frequency with physical fitness through BMI. Model fit indices were evaluated against established criteria (Figures 3, 4). Model fit indices were evaluated against established criteria (Figures 3, 4).

**Figure 3:**
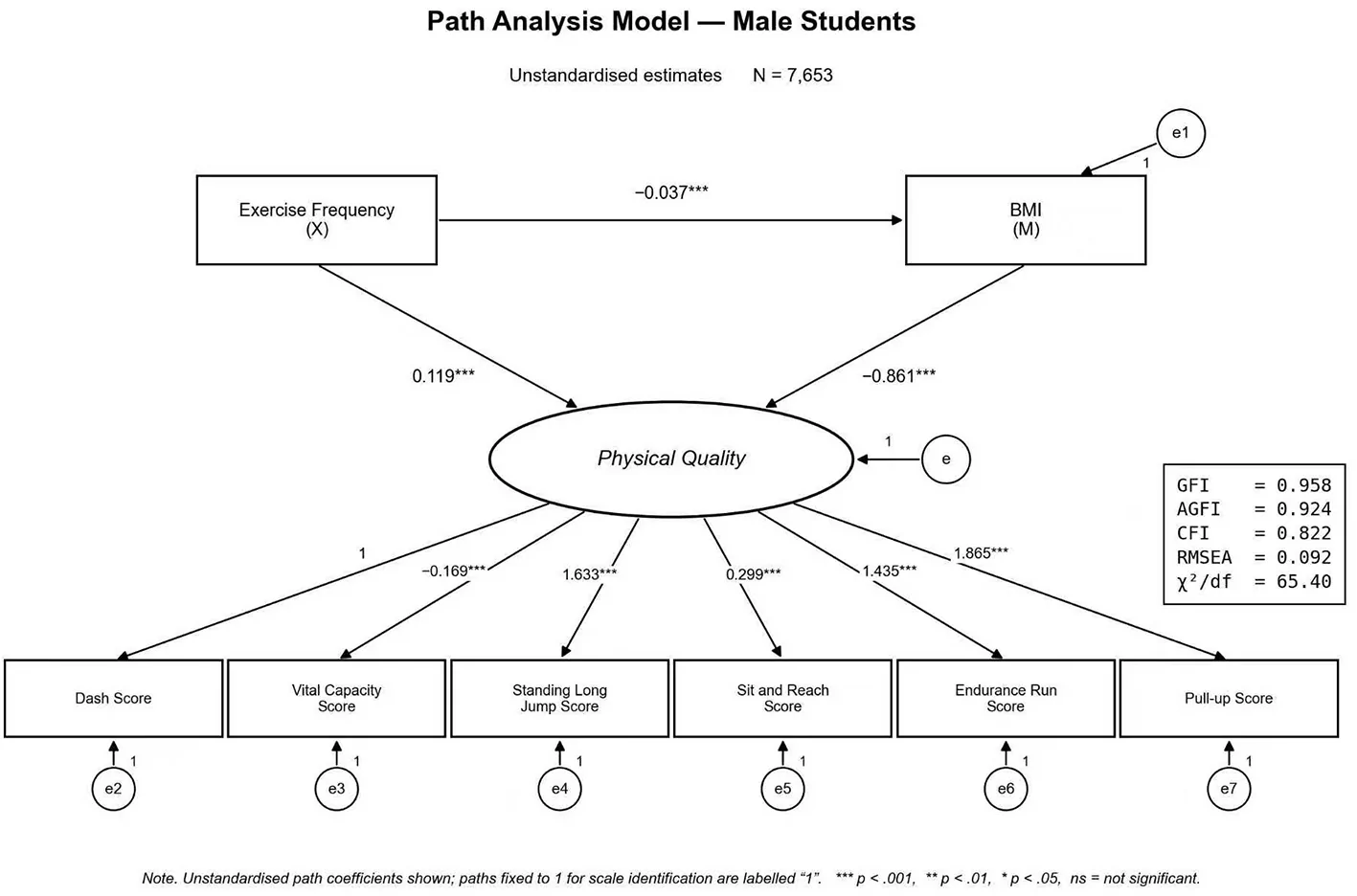
Model fit indices for male participants (*n* = 7,653): *GFI* = 0.958, *AGFI* = 0.924, *CFI* = 0.822, *RMSEA* = 0.092, and χ^2^/*df* = 65.40.

**Figure 4:**
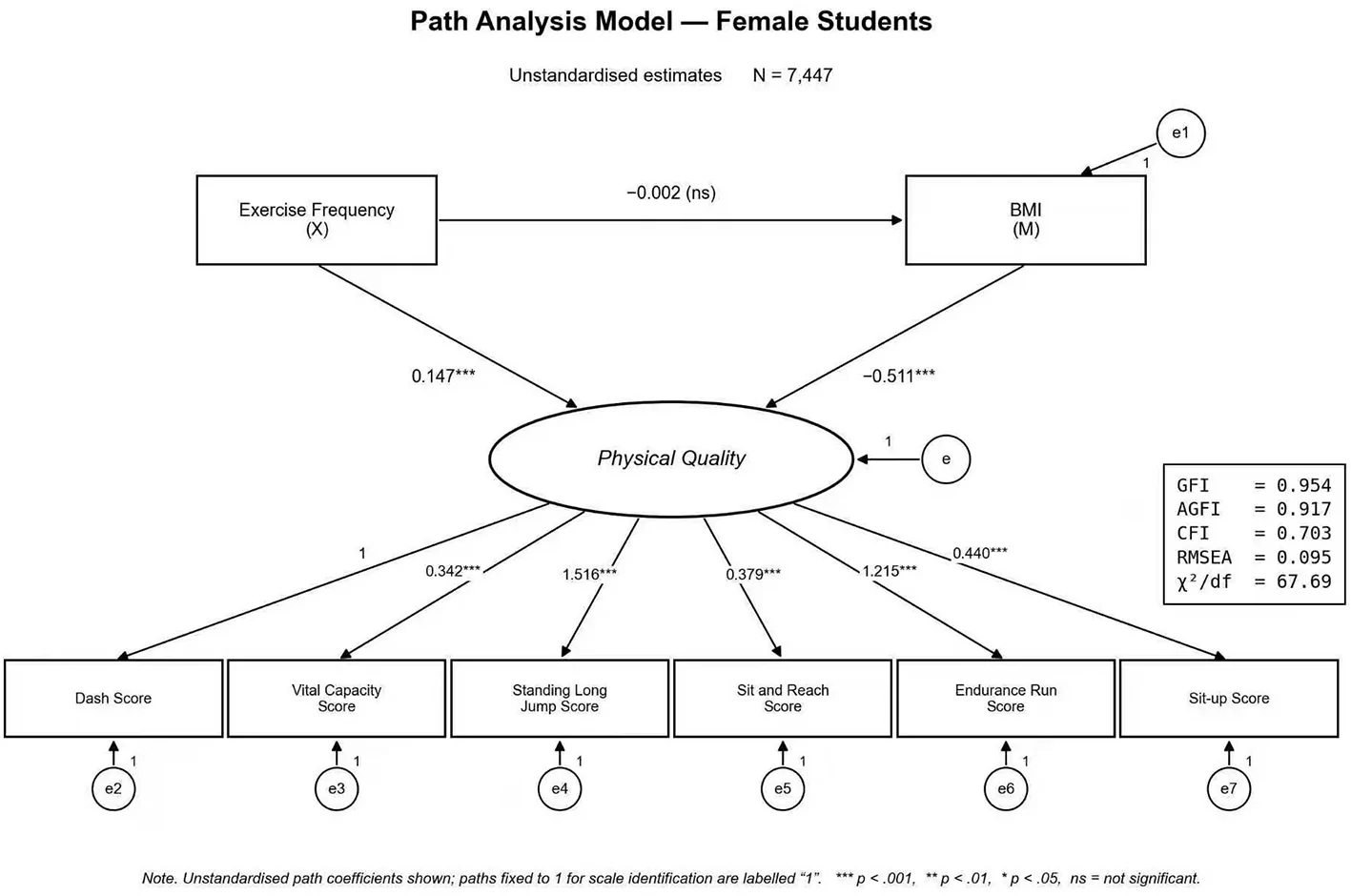
Model fit indices for female participants (*n* = 7,447): *GFI* = 0.954, *AGFI* = 0.917, *CFI* = 0.703, *RMSEA* = 0.095, and χ^2^/*df* = 67.69.

For the male sample comprising 7,653 participants, the Goodness-of-Fit Index was 0.958, the Adjusted Goodness-of-Fit Index was 0.924, the Comparative Fit Index was 0.822, the Root Mean Square Error of Approximation was 0.092, and the chi-square to degrees-of-freedom ratio was 65.40.

For the female sample comprising 7,447 participants, the Goodness-of-Fit Index was 0.954, the Adjusted Goodness-of-Fit Index was 0.917, the Comparative Fit Index was 0.703, the Root Mean Square Error of Approximation was 0.095, and the chi-square to degrees-of-freedom ratio was 67.69.

The GFI and AGFI values for both sexes exceeded the conventional acceptable threshold of 0.90, with the male GFI approaching the excellent threshold of 0.95. However, the CFI fell below 0.90 in both models, and the RMSEA exceeded the recommended cutoff of 0.08, indicating that the proposed path models provided only a moderate representation of the observed data structure. These fit indices suggest that the model-data match is suboptimal. The gender-specific associational-mediation results derived from path analysis should be regarded as exploratory findings rather than definitive conclusions. We did not perform extensive *post-hoc* model respecification to avoid the risk of p-hacking. Accordingly, substantial caution must be exercised when interpreting all path coefficients and corresponding significance values. These associational-mediation outputs are exploratory and do not represent the primary confirmatory finding of this study; the main empirical finding originates from ANOVA and restricted cubic spline analyses.

### Path analysis: male sample

The path analysis results for the male sample of 7,653 participants revealed several significant pathways. Exercise frequency demonstrated a significant negative direct effect on BMI, with a standardized path estimate of negative 0.051, an unstandardized estimate of negative 0.037, a standard error of 0.008, a critical ratio of negative 4.490, and a *p*-value less than 0.001. This indicates that higher habitual exercise frequency was associated with lower BMI among male students (Table 3).

**Table 3:** Path model analysis results for the male sample (*N* = 7,653).

Path	Standardized estimate	Unstandardized estimate	S.E.	C.R.	*p*
Exercise_frequency → BMI	−0.051	−0.037	0.008	−4.490	< 0.001
Exercise_frequency → Physical quality	0.099	0.119	0.015	7.897	< 0.001
BMI → Physical quality	−0.522	−0.861	0.025	−34.937	< 0.001
Physical quality → Dash_Score	0.626	1.000	—	—	—
Physical quality → Vital_Capacity_Score	−0.085	−0.169	0.027	−6.231	< 0.001
Physical quality → Standing_Long_Jump_Score	0.549	1.633	0.048	34.261	< 0.001
Physical quality → Sit_and_Reach_Score	0.136	0.299	0.030	9.939	< 0.001
Physical quality → Endurance_Run_Score	0.631	1.435	0.039	36.988	< 0.001
Physical quality → Pull_up_Score	0.512	1.865	0.057	32.669	< 0.001

Exercise frequency also exhibited a significant positive direct associational pattern with physical quality, independent of its association via BMI. The standardized estimate was 0.099, the unstandardized estimate was 0.119, the standard error was 0.015, the critical ratio was 7.897, and the *p*-value was less than 0.001.

BMI demonstrated a strong negative effect on physical quality, with a standardized estimate of negative 0.522, an unstandardized estimate of negative 0.861, a standard error of 0.025, a critical ratio of negative 34.937, and a *p*-value less than 0.001. This suggests that elevated BMI was substantially associated with poorer overall physical fitness in males.

Regarding the factor loadings of observed indicators on the physical quality latent variable, all loadings were statistically significant at the *p* less than 0.001 level. The dash score served as the reference indicator with its loading fixed to 1.0 for scale identification, and therefore no standard error, critical ratio, or *p*-value was estimated for this path. The vital capacity score loaded negatively onto physical quality with a standardized estimate of negative 0.085, an unstandardized estimate of negative 0.169, a standard error of 0.027, and a critical ratio of negative 6.231. The standing long jump score showed a strong positive loading with a standardized estimate of 0.549, an unstandardized estimate of 1.633, a standard error of 0.048, and a critical ratio of 34.261. The sit-and-reach score loaded positively with a standardized estimate of 0.136, an unstandardized estimate of 0.299, a standard error of 0.030, and a critical ratio of 9.939. The endurance run score exhibited the strongest standardized loading at 0.631, with an unstandardized estimate of 1.435, a standard error of 0.039, and a critical ratio of 36.988. The pull-up score demonstrated a standardized loading of 0.512, an unstandardized estimate of 1.865, a standard error of 0.057, and a critical ratio of 32.669. Notably, vital capacity exhibited a statistically significant negative factor loading on the latent physical-quality construct in the male sample, which appears physiologically counter-intuitive. This unexpected finding suggests that vital-capacity score may serve as an imperfect indicator for the latent physical-fitness construct among male university students, or it may reflect partial model-misspecification. This anomaly should be considered when interpreting outputs from the present latent-variable model. This counter-intuitive negative loading raises concerns over construct validity and measurement-model specification for male participants. We did not fit multiple alternative measurement-model specifications to avoid *post-hoc* p-hacking risk. Therefore, construct validity of this latent physical-quality variable cannot be fully guaranteed in the male subgroup, and all latent-variable-derived outputs for males should be interpreted with great caution.

The path diagram for male students visually represented these relationships. Exercise frequency, positioned as the exogenous variable on the left, directed a path to BMI with a coefficient of negative 0.037 marked with triple asterisks, and a direct path to the physical quality oval with a coefficient of 0.119 also marked with triple asterisks. BMI, positioned as the mediator, directed a path to physical quality with a coefficient of negative 0.861 marked with triple asterisks. The physical quality latent variable, represented as an oval in the center, directed paths to six rectangular observed indicators arranged below: dash score with a fixed loading of 1, vital capacity score with negative 0.169, standing long jump score with 1.633, sit-and-reach score with 0.299, endurance run score with 1.435, and pull-up score with 1.865. Error terms were attached to BMI and each observed indicator. Model fit statistics were displayed in a box on the right side of the diagram, showing GFI at 0.958, AGFI at 0.924, CFI at 0.822, RMSEA at 0.092, and chi-square over degrees of freedom at 65.40.

### Path analysis: female sample

The path analysis results for the female sample of 7,447 participants revealed both convergent and divergent patterns compared with the male sample. Exercise frequency did not significantly predict BMI in females, with a standardized estimate of negative 0.003, an unstandardized estimate of negative 0.002, a standard error of 0.009, a critical ratio of negative 0.217, and a *p*-value of 0.828. This lack of significance indicates that habitual exercise frequency was not associated with BMI variation in this female cohort (Table 4).

**Table 4:** Path model analysis results for the female sample (*N* = 7,447).

Path	Standardized estimate	Unstandardized estimate	S.E.	C.R.	*p*
Exercise_frequency → BMI	−0.003	−0.002	0.009	−0.217	0.828 ns
Exercise_frequency → Physical quality	0.097	0.147	0.022	6.819	< 0.001
BMI → Physical quality	−0.258	−0.511	0.029	−17.406	< 0.001
Physical quality → Dash_Score	0.648	1.000	—	—	—
Physical quality → Vital_Capacity_Score	0.149	0.342	0.034	10.132	< 0.001
Physical quality → Standing_Long_Jump_Score	0.595	1.516	0.052	29.429	< 0.001
Physical quality → Sit_and_Reach_Score	0.186	0.379	0.030	12.523	< 0.001
Physical quality → Endurance_Run_Score	0.539	1.215	0.042	28.839	< 0.001
Physical quality → Sit_up_Score	0.202	0.440	0.033	13.542	< 0.001

However, exercise frequency retained a significant positive direct associational pattern with physical quality, with a standardized estimate of 0.097, an unstandardized estimate of 0.147, a standard error of 0.022, a critical ratio of 6.819, and a *p*-value less than 0.001.

BMI demonstrated a significant negative effect on physical quality, with a standardized estimate of negative 0.258, an unstandardized estimate of negative 0.511, a standard error of 0.029, a critical ratio of negative 17.406, and a *p*-value less than 0.001. The magnitude of this effect was notably attenuated compared with that observed in the male sample.

All factor loadings of observed indicators on physical quality were positive and statistically significant at the *p* less than 0.001 level. The dash score served as the reference indicator with its loading fixed to 1.0. The vital capacity score loaded positively with a standardized estimate of 0.149, an unstandardized estimate of 0.342, a standard error of 0.034, and a critical ratio of 10.132. The standing long jump score showed the strongest standardized loading at 0.595, with an unstandardized estimate of 1.516, a standard error of 0.052, and a critical ratio of 29.429. The sit-and-reach score loaded with a standardized estimate of 0.186, an unstandardized estimate of 0.379, a standard error of 0.030, and a critical ratio of 12.523. The endurance run score exhibited a standardized loading of 0.539, an unstandardized estimate of 1.215, a standard error of 0.042, and a critical ratio of 28.839. The sit-up score demonstrated a standardized loading of 0.202, an unstandardized estimate of 0.440, a standard error of 0.033, and a critical ratio of 13.542.

The path diagram for female students followed a similar structural layout to the male model. Exercise frequency directed a path to BMI with a coefficient of negative 0.002 marked as non-significant, and a direct path to physical quality with a coefficient of 0.147 marked with triple asterisks. BMI directed a path to physical quality with a coefficient of negative 0.511 marked with triple asterisks. The physical quality latent variable directed paths to six observed indicators: dash score with a fixed loading of 1, vital capacity score with 0.342, standing long jump score with 1.516, sit-and-reach score with 0.379, endurance run score with 1.215, and sit-up score with 0.440. Model fit statistics displayed in the diagram showed GFI at 0.954, AGFI at 0.917, CFI at 0.703, RMSEA at 0.095, and chi-square over degrees of freedom at 67.69.

### Summary of sex-specific pathway findings

The path analysis revealed both convergent and divergent patterns between male and female samples. Convergently, exercise frequency demonstrated a significant positive direct associational pattern with physical quality in both sexes, independent of BMI. This suggests that regular exercise is correlated with favorable fitness outcomes through potential pathways other than, or in addition to, body-composition-related correlates. Divergently, the associational mediation chain of exercise frequency-BMI-physical quality was supported only in males. In females, exercise frequency was unrelated to BMI, thereby precluding BMI-mediated indirect effects on physical quality. Furthermore, numerically larger negative coefficient magnitude for BMI predicting physical quality was observed among male participants (standardized coefficient −0.522) compared with female participants (standardized coefficient −0.258). This is only a numerical exploratory observation and cannot be interpreted as statistically proven sex difference, given absent measurement invariance testing and sex-specific strength-test items. This sex difference suggests that BMI may function as a more critical determinant of overall physical fitness in male university students, whereas in females, the beneficial effects of exercise on physical quality operate largely through non-BMI pathways.

## Discussion

The present findings align with the growing body of literature indicating that the relationship between exercise frequency and physical fitness may not be strictly linear, particularly in young adult populations. While engaging in physical activity yields substantial health benefits, with even a moderate increase from a sedentary state leading to significant improvements, the observation that further increases from moderate to high frequency do not yield additional significant gains resonates with intervention studies. For instance, a meta-analysis on adolescent physical fitness concluded that the most effective regimen involved exercise three times per week, with higher frequencies not conferring proportional benefits (25). Similarly, in older adults, concurrent exercise training performed three times per week increased serum BDNF levels to a similar extent as five times per week, suggesting a ceiling effect (10). A real-life program for type 2 diabetes patients showed negligible differences between a standard program and an alternative program with lower frequency (26) and research on complex contrast training found that two sessions per week provided effects largely similar to three sessions per week in active adult males (8). These data extend these observations to a large-scale sample of Chinese university students, reinforcing that the most pronounced benefits accrue from transitioning from a low to a moderate level of exercise engagement.

Path analysis further revealed sex-specific associational patterns underlying this observed relationship. In males, exercise frequency demonstrated a significant negative direct association with BMI, indicating higher habitual activity was associated with lower adiposity, which was concurrently correlated with better physical fitness, as elevated BMI is a well-established negative predictor of fitness performance (27, 28). Conversely, in females, exercise frequency did not significantly predict BMI, aligning with studies observing a weaker association between physical activity and BMI in females, potentially due to sex differences in body composition where fat mass percentage is more strongly associated with fitness levels. The significant direct effect of exercise frequency on physical fitness, independent of BMI, was observed in both sexes, suggesting benefits through mechanisms beyond weight control, such as neuromuscular and metabolic adaptations (29, 30). Furthermore, the negative effect of BMI on physical fitness was stronger in males, we observed numerically stronger correlation magnitude between BMI and physical-fitness among male participants in this sample, possibly reflecting differences in lean mass influence. Thus, while BMI presented a key associational-mediation statistical pattern in males, this pattern was not supported in females, consistent with findings that physical activity is correlated with better fitness in females via non-BMI-related statistical associational patterns.

In the context of college students, exercise frequency has been identified as a key factor influencing physical fitness levels (31). The present study's core contribution is the validation, using a large sample of 15,100 university students and objectively recorded exercise data from a mobile application, of this complex relationship, offering an empirical foundation for designing precise interventions in higher education. Previous research has established that adolescents who engage in regular physical activity exhibit superior fitness (32) with meta-analyses noting a dose-response association but highlighting the need to identify optimal thresholds (33, 34). Our findings build upon this by demonstrating that beneficial effects are most pronounced when moving from low to moderate frequency, aligning with research showing significant gains in health parameters at moderate activity levels (35). Similarly, intervention studies in educational settings support that a moderate threshold is particularly impactful. The observed plateau beyond moderate frequency suggests that a feasible goal of three to four sessions per week may be more effective than striving for more frequent, but potentially less sustainable, routines.

The observed pattern of diminishing returns aligns with the threshold effect described in prior epidemiologic research, where the greatest gains are achieved at moderate activity levels with progressively smaller improvements thereafter. Such a non-linear relationship has been observed in other contexts; for instance, in the association between physical activity and depression among weekend catch-up sleepers, moderate activity offered maximal mental health benefits (36) and a study on exercise and breast cancer recurrence found a linear reduction in risk up to approximately 25 MET-h/wk, with no additional benefit beyond this threshold (37). This pattern suggests a generalizable principle wherein a certain dose yields optimal returns for specific outcomes.

We offer several literature-based speculative hypotheses to interpret observed sex-specific patterns; note that these potential mechanisms cannot be directly verified within our dataset since relevant biological and behavioral variables were not measured. The sex-specific discrepancy in the relationship between exercise frequency and body composition, with a significant inverse association only in males, aligns with research suggesting males may be more physiologically responsive to exercise-induced changes in adiposity, potentially due to androgenic regulation facilitating greater fat oxidation. The absence of a significant link in females may reflect estrogen's moderating influence on lipid metabolism or sex differences in habitual activity patterns, as women often engage in less vigorous exercise (38)Since our study recorded exercise frequency without accounting for intensity, female participants may have engaged in sessions of lower metabolic demand, attenuating the dose-response effect on BMI, as exercise intensity is a critical determinant of adiposity reduction, particularly in females. Additionally, gender-specific differences in post-exercise dietary intake could further obscure this relationship. These findings underscore the complexity of the exercise-body composition nexus and highlight the need for future research to incorporate detailed measures of intensity, energy expenditure, and dietary patterns.

The use of objectively measured exercise frequency from a mobile application offers distinct methodological advantages over self-report questionnaires, which are susceptible to recall bias and misclassification. Objective recordings from digital platforms provide a more reliable assessment of habitual patterns, enhancing internal validity and aligning with calls for rigorous exposure measurement. However, this advantage comes with a trade-off: the exclusive focus on frequency precludes capture of other critical dimensions, including intensity, duration, and modality, as the benefits of resistance training differ from aerobic exercise. Furthermore, the inability to account for total energy expenditure or differentiate between structured exercise and non-exercise physical activity limits comprehensiveness, potentially introducing residual confounding from factors known to influence body composition and fitness (39).

The present investigation systematically delineates sex-specific pathways through which exercise frequency influences physical fitness, with a focus on BMI's mediating role. This approach extends prior research that often examined these relationships in isolation (40). While previous studies have established that physical activity is associated with better fitness and lower BMI, the mechanisms, especially the mediating role of BMI and sex-specific differences, have been less clearly defined using comprehensive structural equation models in a large, homogenous population. Our findings support a significant direct pathway from exercise frequency to physical fitness, independent of BMI, in both sexes, aligning with evidence that benefits are not solely mediated by body composition but also through other physiological adaptations.

These findings carry important implications for public health policy and physical education programs. The threshold effect suggests that university sports policies should strategically prioritize transitioning students from an inactive state (≤ 2 sessions/week) to a moderately active state (3-4 sessions/week), a more pragmatic approach than setting unattainably high goals, which may lead to dropout. This aligns with the well-documented benefits of moderate activity for improving health-related quality of life among college populations (41) and broader strategies focusing on reducing sedentary behavior.

The finding that the overall fitness score exhibited the most pronounced differences across exercise frequency groups underscores the utility of a composite metric in university fitness surveillance. Comprehensive fitness indices more effectively capture the multifaceted nature of physical health (42) as seen in workplace wellness programs showing improvements in composite functional movement scores (42). Research on preschool children has highlighted that principal component analysis can reveal distinct performance profiles (43) and in military populations, holistic assessments are recognized as superior for gauging readiness. The observed threshold effect aligns with research indicating that the association between activity volume and health outcomes is not always linear. Therefore, prioritizing an overall fitness score in institutional assessments may provide a more accurate metric for evaluating exercise interventions.

Several methodological considerations warrant discussion. The cross-sectional design precludes causal inference; reverse causation is plausible, as individuals with greater fitness may be more predisposed to engage in higher exercise frequency, a pattern observed in older adults. Residual confounding from unmeasured variables, such as socioeconomic status (44), dietary behaviors, and sleep quality, remains a notable concern even for campus-dwelling student samples may also influence the observed associations. To establish causal pathways, future research should prioritize longitudinal designs or randomized controlled trials.

The path analysis models demonstrated moderate fit, with CFI values below 0.90 and RMSEA values exceeding 0.08, indicating an incomplete representation of the underlying mechanisms. This aligns with other studies where achieving excellent fit requires a more comprehensive set of predictors. The omission of potentially critical variables, such as emotional demands (45) training intensity (46) muscle strength, and psychosocial factors like self-efficacy (47)or social support, may explain the moderate fit. Future iterations should incorporate these variables, potentially using multi-mediator or moderated mediation models, and treat sex as a moderator within a multigroup analysis framework for more rigorous comparisons.

The categorization of exercise frequency into groups based on a priori thresholds (≤ 2, 3–4, and ≥5 sessions per week) does not account for session duration or intensity, a notable limitation as total volume is a composite of these factors. Within the high-frequency group, there could be substantial heterogeneity in actual load, and measures that fail to integrate intensity can misclassify true exposure (48). Health benefits are more strongly linked to total energy expenditure, often expressed as MET-minutes per week (49) or time spent in moderate-to-vigorous activity (50). Future investigations should integrate a more comprehensive measure of exercise load and use continuous exposure variables to more precisely test for non-linear associations and identify thresholds.

Several limitations should be acknowledged. The sample was exclusively composed of undergraduate students from a single Chinese university, constraining generalizability to broader populations. The cross-sectional design precludes causal inferences, and while behavioral assessments are valuable, they may not capture the full spectrum of neuroplastic changes. Future investigations should include participants from multiple institutions and extend the age range, particularly those with mild cognitive impairment. Incorporating additional fitness domains, such as agility and coordination, and considering exercise type or timing relative to injury would provide a more comprehensive understanding. Given the link between exercise-induced cortical excitability and cognitive performance (51) future work should explore underlying neurobiological mechanisms (52).

## Conclusion

Based on a large sample of 15,100 Chinese university students with objectively-captured exercise records, this cross-sectional work explored the associational patterns linking weekly exercise frequency to multi-dimensional comprehensive physical fitness, alongside sex-specific associational-mediation roles of BMI. Restricted cubic spline regression supported a threshold-like non-linear associational pattern: pronounced fitness improvements were observed when participants shifted from low-frequency (≤ 2 sessions per week) to moderate-frequency (3-4 sessions per week) exercise, while marginal fitness improvements became attenuated in this sample at higher exercise frequency. Such a pattern was consistent across most physical-fitness indicators examined in ANOVA comparisons. As purely exploratory observations from sub-optimal-fit path-analysis models, associational-mediation patterns linked to BMI were only suggested among male students, while among female students, only the direct association between exercise frequency and physical fitness was observable, with no detectable associational-mediation effect through BMI. Importantly, positive direct associations bet ween exercise frequency and physical fitness existed for both sexes independent of BMI-related correlates, illustrating that physical-fitness gains from exercise are not fully explained by body-composition-related correlates alone and that alternative physiological pathways should be considered when interpreting exercise-fitness relationships.

These empirical observations carry clear practical value for physical-education management and public-health promotion within higher-education institutions. Based on cross-sectional associational observations from this sample, students with recorded moderate exercise frequency (3-4 sessions per week) tended to display better comprehensive physical fitness compared with low-frequency peers. This correlational finding cannot serve as definitive exercise prescription, as it does not prove that assigning 3-4 sessions per week will causally improve fitness, nor does it rule out potential fitness benefits for participants exercising ≥5 sessions weekly. Universities may consider guiding physically inactive students toward moderate exercise levels, while avoiding over-emphasis on extremely high weekly exercise volume which may lead to poor adherence and high dropout risk. Sex-differentiated intervention strategies deserve full consideration in practical campus work. These exploratory findings generate preliminary hypotheses for future research: it may be worthwhile for future studies to test whether BMI-related health management could bring additional fitness-related benefits for male college students, and whether non-BMI-related pathways deserve more attention for female students. These potential sex-differentiated considerations cannot serve as evidence-based intervention guidance in the present cross-sectional study. In addition, composite overall-fitness scores demonstrated superior sensitivity in reflecting group-level differences across exercise-frequency strata, which supports the wider adoption of multi-dimensional composite fitness indicators for routine student physical-health surveillance and the evaluation of campus physical-activity programmes. When designing campus exercise guidance, educators should avoid overemphasizing excessive exercise frequency and pay more attention to helping students form stable, sustainable moderate-intensity exercise habits.

Interpretation of the present findings should consider several inherent study constraints: This cross-sectional study cannot establish temporal ordering or causal relationships between exercise frequency and physical-fitness outcomes, and reverse causation cannot be excluded since individuals with better baseline fitness may be more inclined to engage in regular exercise. Although restricted cubic spline analysis supported our grouping thresholds, path-analysis models for male and female subgroups exhibited sub-optimal CFI and RMSEA fit indices; therefore all observed associational-mediation results should be interpreted as exploratory rather than definitive conclusions, and we avoided *post-hoc* model modification to prevent P-hacking. BMI served as the only available body-composition indicator in this dataset and cannot differentiate fat mass from lean muscle mass, creating interpretive uncertainty particularly among physically active young adults, while body fat percentage and waist circumference data were unavailable for supplementary validation. Key covariates including dietary patterns, sleep quality and socioeconomic status were not collected, so residual confounding could not be statistically adjusted for, even though participants were enrolled from campuses under relatively unified management. Exercise frequency data originated from the Chuanggao Sports APP without published peer-reviewed reliability or validity evidence, and the dataset only recorded exercise occurrence frequency without objective exercise-intensity metrics. Given different upper-body/core-strength test items for males and females, formal multigroup measurement invariance testing was not performed, restricting rigorous quantitative comparison of factor-loading and path-coefficient magnitudes across sexes. Finally, the sample was recruited exclusively from a single university, which limits the generalizability of these associational patterns to broader Chinese undergraduate populations. Findings are primarily generalizable to undergraduate students under similar campus-managed university settings; caution should be taken when generalizing to broader young-adult populations. Further longitudinal multi-site studies incorporating detailed covariate, exercise-intensity and body-composition measurements are needed to replicate and extend the present findings.

## Data Availability

The raw data supporting the conclusions of this article will be made available by the authors, without undue reservation.
